# Draft Genome Sequences of Biosafety Level 2 Opportunistic Pathogens Isolated from the Environmental Surfaces of the International Space Station

**DOI:** 10.1128/genomeA.01263-16

**Published:** 2016-12-29

**Authors:** Aleksandra Checinska Sielaff, Nitin K. Singh, Jonathan E. Allen, James Thissen, Crystal Jaing, Kasthuri Venkateswaran

**Affiliations:** a Jet Propulsion Laboratory, California Institute of Technology, Pasadena, California, USA; b Lawrence Livermore National Laboratory, Livermore, California, USA

## Abstract

The draft genome sequences of 20 biosafety level 2 (BSL-2) opportunistic pathogens isolated from the environmental surfaces of the International Space Station (ISS) were presented. These genomic sequences will help in understanding the influence of microgravity on the pathogenicity and virulence of these strains when compared with Earth strains.

## GENOME ANNOUNCEMENT

In an on-going Microbial Observatory experiment on the International Space Station (ISS), multiple biosafety level 2 (BSL-2) bacterial isolates were isolated, identified, and whole-genome sequences (WGS) were generated. The genomic data enables the determination of the microgravity influence on pathogenicity and virulence in these microorganisms by comparison to type strains of the corresponding species.

*Acinetobacter pittii* is a nonmotile coccobacilli isolated for the first time from cerebrospinal fluid (1). Multiple strains of *A. pitti* were isolated from the cupola area. The IIF1SW-P1 was resistant to cefazolin, cefoxitin, oxacillin, penicillin, and rifampin.

Two multidrug-resistant *Enterobacter* sp. isolates were found in the waste and hygiene compartment (WHC) location. Species of *Enterobacter cloacae* complex (Ecc) are commonly found in the environment, but are of high clinical significance (2).

*Pantoea conspicua* was originally isolated from human blood (3). This was the second most prevalent species, and was only found in one location during two different flight samplings. *P. conspicua* isolates were resistant to erythromycin, oxacillin, penicillin, and rifampin.

*Staphylococcus* isolates were the most prevalent from ISS surfaces. *Staphylococcus aureus* was the most abundant in all ISS locations. Although this species is a common human commensal (4), it causes various types of minor skin infections, bacteremia, or scalded skin syndrome, especially in immunocompromised individuals (5). In this study, some of the isolates were found to be resistant to erythromycin (IF4SW-P1, IF7SW-P3) and most of the isolates were resistant to penicillin. A few isolates acquired rifampin resistance during the study (RA isolates).

*Staphylococcus haemolyticus* and *Staphylococcus hominis* belong to coagulase-negative staphylococci (6, 7). *S. hominis* IIF4SC-B9 was resistant to penicillin and erythromycin, but *S. haemolyticus* IIF2SW-P5 was susceptible to these antibiotics. All three species are reported to be methicillin resistant by acquiring the staphylococcal cassette chromosome *mec* (SCC*mec*) (8), but the methicillin-resistant phenotype was not observed.

In this study, the draft genomes sequences of 20 strains from the ISS were obtained. WGS sequencing was performed on an Illumina NextSeq instrument with a paired-end module. The A5 assembly pipeline version 20150522 was used to generate draft assemblies applying the default parameter settings (9) and annotated with the help of the Rapid Annotations using Subsystems Technology (RAST) (10). Table 1 summarizes assembly statistics (number of contigs, total genome size, *N*_50_ size, median coverage, G+C percentage, error corrected reads used for assembly, and number of coding sequences). The raw reads were in the range of 24 to 82 Mbp per genome. The G+C content was in the range of 31.5 to 38.7% for *Staphylococcus* species and *A. pittii*; for other strains the G+C contents were 55.2 to 55.8%. The subsystem features created using RAST for all 20 strains are depicted in Table 1.

**TABLE 1:** Statistics summary for the 20 draft ISS BSL-2 bacterial genome sequences

Strain	NCBI accession no.	Isolation location	No. of contigs	Genome size (bp)	*N*_50_ (bp)	Median coverage	G+C content (%)	Error corrected reads	Coding sequences
*A. pittii* IIF1SW-P1	MIZX00000000	Port panel next to cupola	150	4,041,255	144,373	799	38.7	25,486,884	3,821
*Enterobacter* sp. IF2SW-B1	MJAA00000000	WHC^a^	437	5,097,299	306,837	686	55.2	24,992,043	4,671
*Enterobacter* sp. IF2SW-P2	MJAB00000000	WHC^a^	230	4,974,814	298,912	850	55.8	30,618,796	4,629
*P. conspicua* IF5SW-P1	MIZY00000000	Node 1 overhead 4	280	5,126,609	216,776	797	55.6	34,104,170	4,852
*S. aureus* IF4SW-P1	MIZH00000000	Dining table	498	2,980,137	64,789	3,695	32.7	76,859,228	2,733
*S. aureus* IF6SW-P2	MIZI00000000	PMM port 1^b^	204	2,836,553	355,893	2,578	32.8	51,467,673	2,657
*S. aureus* IF6SW-P2-RA	MIZK00000000	PMM port 1^b^	228	2,845,178	295,897	2,740	32.8	55,167,977	2,659
*S. aureus* IF6SW-P3A	MIZJ00000000	PMM port 1^b^	276	2,868,506	232,680	2,254	32.8	46,555,897	2,694
*S. aureus* IF6SW-P3A-RA	MIZL00000000	PMM port 1^b^	257	2,861,821	264,865	2,733	32.8	47,711,605	2,690
*S. aureus* IF7SW-P3	MIZM00000000	Lab overhead 3	452	2,951,917	52,140	3,487	32.8	71,062,021	2,738
*S. aureus* IIF6SW-P2	MIZN00000000	PMM port 1^b^	312	2,884,460	96,689	3,324	32.8	67,792,619	2,730
*S. aureus* IIF6SW-P2-RA	MIZR00000000	PMM port 1^b^	192	2,835,299	325,968	2,021	32.8	42,250,883	2,655
*S. aureus* IIF6SW-P3	MIZO00000000	PMM port 1^b^	194	2,837,901	467,825	2,638	32.8	54,334,144	2,657
*S. aureus* IIF6SW-P3-RA	MIZS00000000	PMM port 1^b^	217	2,841,156	411,108	2,272	32.8	47,711,605	2,656
*S. aureus* IIF8SW-P1	MIZP00000000	Port crew quarters bump-out exterior aft wall	143	2,817,304	425,858	2,409	32.8	49,197,886	2,637
*S. aureus* IIF8SW-P1-RA	MIZT00000000	Port crew quarters bump-out exterior aft wall	201	2,848,005	526,364	1,834	32.7	39,316,061	2,653
*S. aureus* IIF8SW-P2	MIZQ00000000	Port crew quarters bump-out exterior aft wall	194	2,830,972	329,726	2,557	32.8	51,625,221	2,650
*S. aureus* IIF8SW-P2-RA	MIZU00000000	Port crew quarters bump-out exterior aft wall	141	2,822,756	526,364	2,014	32.8	42,704,251	2,642
*S. haemolyticus* IIF2SW-P5	MIZW00000000	WHC^a^	567	2,680,722	48,308	2,945	33.1	56,836,461	2,518
*S. hominis* IIF4SC-B9	MIZV00000000	Dining table	508	2,420,684	79,555	3,738	31.5	61,283,456	2,301

^a^ WHC, waste and hygiene compartment.

^b^ PMM port 1, permanent multipurpose module.

### Accession number(s).

The WGS data were deposited at DDBL/EMBL/GenBank under the accession no. listed in Table 1 and at the NASA GeneLab system (GLDS-67; https://genelab-data.ndc.nasa.gov/genelab/accession/GLDS-67/##). The version described in this paper is the first version. The strains were deposited in the USDA Agricultural Research Station (NRRL) and German culture collections.
